# Association between strategic differentiation and firm leverage manipulation: Empirical evidence from China

**DOI:** 10.3389/fpsyg.2022.1013257

**Published:** 2023-01-04

**Authors:** Feng Niu, Jiayi Wang, Wunhong Su

**Affiliations:** School of Accounting, Hangzhou Dianzi University, Hangzhou, Zhejiang Province, China

**Keywords:** strategic differentiation, leverage manipulation, financing constraints, deleveraging policy, China

## Abstract

**Introduction:**

Based on the context of the implementation of China’s comprehensive deleveraging policy, this study explores the impact of firm strategic differentiation on the emergence of leverage manipulation behavior.

**Methodology:**

The A-share listed firms in Shanghai and Shenzhen from 2007 to 2020 were used as the research objects, and the data were processed using Stata software.

**Results:**

The greater the degree of strategic differentiation, the higher the likelihood of firm leverage manipulation. The effect of strategic differentiation on leverage manipulation is more significant when firms are under short-term debt service pressure. Auditor industry expertise can weaken the positive relationship between strategic differentiation and firm leverage manipulation. Further results show that the degree of strategic differentiation increases the likelihood of firm leverage manipulation by increasing the degree of financing constraints of the firm.

**Conclusion:**

This study enriches the literature on leverage manipulation, provides empirical evidence on the economic consequences of strategic differentiation, and has important implications for the precise implementation of deleveraging policies by relevant government departments, as well as a reference for external investors’ decisions.

## Introduction

In response to the 2008 financial crisis, China adopted economic stimulus policies. According to the data released by the center of the national balance sheet (CNBS) of the Chinese Academy of Social Sciences, the leverage ratio of China’s non-financial firms surged from 95.2% at the end of 2008 to 160.4% in March 2017. By the end of 2018, the leverage ratio of China’s non-financial firms (151.6%) was much higher than that of developed economies such as Japan (102.6%), the eurozone (105%), and the United States (74.4%) and Germany (56.7%). Moreover, in the past 2 years, with the outbreak of COVID-19, many firms began a new round of leverage to maintain their operations, further exacerbating the upward pressure on leverage.^1^ According to the data released by the People’s Bank of China on the financing scale, the loans issued to the real economy in the first half of 2020 increased by 12.09 trillion yuan, an increase of 2.42 trillion Yuan year-on-year. In addition, the leverage ratio of the non-financial firms reached 165.2% in June 2020, a new historical peak. The high leverage of firms not only greatly increases the debt risk faced by firms but also is likely to trigger further more extensive and serious financial risks in the market. Therefore, effectively reducing firms’ leverage ratio plays an important role in promoting the reduction of China’s total leverage ratio and preventing and resolving major risks (Reinhart and Rogoff, 2011; Jin et al., 2017).^2^

Furthermore, the central government has attached great importance to the severe challenge of high leverage to China’s economic and financial stability. In 2015, the Chinese central government put forward the task of reducing leverage and setting deleveraging as the national strategy, thus officially entering the mandatory deleveraging period. In July 2017, the Chinese central government emphasized deleveraging of state-owned enterprises (SOEs) as the top priority of firm deleveraging. After a year, the Chinese Central Finance and Economics Commission introduced the basic idea of structural deleveraging and issued guidelines. The introduction of this series of policies highlights the Chinese government’s determination to deleverage. The implementation of deleveraging policies has also achieved initial goals. According to data published by the National Institute of Finance and Development (NIFD) in China, the leverage ratio of non-financial firms has fallen to 154.8% by the end of 2021 from a peak of 165.3% in 2020.

An important issue is whether firms steadily achieve their deleveraging goals. Under the influence of multiple factors such as external deleveraging policy pressure, the impact of the epidemic since 2020, and their operations, many firms usually find it difficult to reduce leverage by repaying debt or increasing equity financing and instead choose to manipulate leverage by using nominal equity but real debt, and off-balance sheet liabilities and accounting manipulation to achieve formal deleveraging goals, cater to policies and obtain a more relaxed financing environment (Xu and Lu, 2020). In other words, the decline in firm leverage in recent years is not entirely a true reflection of the effects of deleveraging policies but rather the result of leverage manipulation. However, while it is true that leverage manipulation can formally reduce a firm’s leverage and send a “good” signal to the investors as an urgent need, it is more likely to increase the financial risk of the firm, mislead the judgment of external information users and trigger systemic risk in the capital markets. Therefore, firm leverage manipulation is a thirst quencher, contrary to the original intention of the national deleveraging policy.

Based on this, some studies analyze the factors influencing firm leverage manipulation from the perspectives of controlling shareholders’ equity pledges (Xu et al., 2021), party organization governance (Zhai et al., 2021), institutional investors’ “distraction” (Wu et al., 2022), and local government debt (Rao et al., 2022). Most of these studies discuss various external factors with firm leverage manipulation through financing perspectives. However, firm-specific behavior is mostly endogenous to firm strategy. Strategic positioning determines how firms allocate their resources (Miles et al., 1978), shapes their behavioral decisions, and is a deeper factor affecting leverage manipulation. Typically, firms choose conventional strategic models that increase their legitimacy and reduce uncertainty (Meyer and Rowan, 1977). However, some firms deviate from the industry’s conventional strategic model to gain a competitive advantage. The extent to which this pattern of resource allocation deviates from the industry’s conventional strategic model is called “strategic differentiation” (Finkelstein and Hambrick, 1990).

In other words, an industry in the growth and development process gradually accumulates a kind of industry conventional strategic model representing the overall development direction of the industry. The problem of strategic differentiation arises when a firm deviates from the conventional strategic direction of the industry in its strategic positioning and chooses a strategic model of industry differentiation. A greater degree of deviation helps firms enhance their competitiveness, foster customer loyalty, and improve performance, but it often also means higher costs and greater uncertainty. Thus, strategic differentiation is a “double-edged sword” that can bring excess returns or expose firms to higher operational risks and more stringent financing constraints. Therefore, it is of great practical importance to examine the association between firm strategic differentiation and leverage manipulation and its mechanism to promote the effectiveness of deleveraging policies.

This study investigates the association between strategic differentiation and firm leverage manipulation using data from 2007 to 2020 for listed firms in Shanghai and Shenzhen A-shares in China, following Xu et al. (2020) for the measure of firm leverage manipulation. The results show that the greater strategic differentiation, the more likely a firm is to engage in leverage manipulation. Furthermore, the positive relationship between strategic differentiation and leverage manipulation is more significant for firms with higher short-term debt repayment pressure. Auditor industry expertise weakens the positive relationship between strategic differentiation and leverage manipulation. The mechanism test results suggest that strategic differentiation increases the likelihood of firm leverage manipulation by enhancing firm financing constraints.

The possible contributions of this study are three aspects. First, this study expands and deepens research on the economic consequences of firm strategic differentiation. Most of the existing literature on strategic differentiation is developed based on the income statement, focusing on earnings management and accounting information quality (e.g., Bentley et al., 2012). This study concentrates on leverage manipulation on a balance sheet basis to expand the perspective of strategic differentiation. Second, this study enriches and expands the literature on factors influencing leverage manipulation. Few studies discuss the factors influencing leverage manipulation. After the concept and measurement of leverage manipulation were proposed, only equity pledges, party organization governance, local government debt, and institutional investor “distraction” were addressed (e.g., Xu et al., 2021; Zhai et al., 2021; Rao et al., 2022; Wu et al., 2022). This study presents strategic factors affecting leverage manipulation regarding firms’ strategic differentiation.

Third, this study provides an in-depth analysis of the relationship between strategic differentiation and leverage manipulation from multiple perspectives and clarifies the underlying mechanism of strategic differentiation affecting leverage manipulation. This study examines the heterogeneity of the relationship between strategic differentiation and leverage manipulation from two perspectives: short-term debt repayment pressure and auditor industry expertise, respectively. The results provide a more focused reference for addressing leverage manipulation as a thirst quencher and exploring how strategic differentiation affects leverage manipulation through financing constraints.

The remainder of the paper is organized as follows. The next section demonstrates the literature review and develops research hypotheses. The third section describes the research design. The fourth section presents empirical results. The fifth section illustrates the mechanism test, and the sixth section reports robustness test results. Finally, the seventh section indicates research conclusions and policy recommendations.

## Literature review and hypothesis development

### Literature review

Existing research on the issue of strategic differentiation has focused on the relationship between strategic differentiation and firm performance and financing constraints. For example, Shrader and Simon (1997) argue that firm strategic differentiation reduces operating performance. Goll et al. (2007) demonstrate that strategic differentiation is positively associated with a firm’s operating performance. Literature further documents the effect of differentiation on the volatility of operating performance and concludes that strategic differentiation increases the likelihood of operating volatility and extreme firm performance (e.g., Chen and MacMillan, 1992; Liu and Lee, 2019; Xu and Wang 2022). Further, strategic differentiation inevitably requires firms to make more specialized investments to form specialized assets that can improve their core competitiveness. But this also increases the cost of adjusting assets, making it impossible for firms to reduce the various R&D investments made earlier when they are in a period of operational contraction, leading to the prominent problem of cost stickiness in differentiated firms (e.g., Che and Duan, 2016; Mei and Xiao-Ju, 2017).

Furthermore, Hu and Zheng (2021) conclude that strategic differentiation increases the difficulty of obtaining commercial financing. Firms with few tangible assets and in more competitive industries have more difficulty obtaining commercial credit financing. Strategic differentiation also further increases firms’ default risk through agency costs and operational risk, which leads to an increase in firms’ cost of equity capital, cost of debt financing, and cost of commercial credit financing (e.g., Hiller and Hambrick, 2005; Wang et al., 2017, 2019; Li and Yu, 2021).

In addition, other related studies on strategic differentiation have attracted continued academic attention. For example, some studies find that the firm strategic differentiation affects the quality of firm accounting information (e.g., Bentley et al., 2012), firm technological innovation (e.g., Kim, 2010), and firm tax avoidance (e.g., Pei-Hui et al., 2018). Some studies also explore the relationship between analyst behavior external to the firm (e.g., He and Yin, 2018; Liu and Shi, 2018), auditor decision-making and governance (e.g., Chen, 2015), and the differentiation in firm strategy.

Although firm leverage manipulation has been around for a long time, the academic discussion on leverage manipulation did not form a systematic concept. Xu et al. (2020) establish a comprehensive measure of leverage manipulation using the XLT-LEVM method. As a result, research on firm leverage manipulation has taken a new step forward. Regarding the factors influencing leverage manipulation, literature developed several perspectives, which can be divided into two main aspects: the motivation of firm leverage manipulation and the influence of external governance factors. In terms of the motivation for leverage manipulation, Xu et al. (2021) find that controlling shareholders, who are affected by the risk of control transfer and debt repayment pressure after pledging their equity, would favor leverage manipulation to reduce firm leverage for market capitalization management.

Furthermore, considering that one of the main motives for firm leverage manipulation is to satisfy their own financing needs and reduce financing constraints, some studies, based on the crowding-out effect of local government debt, find that the problem of difficult and expensive financing for firms is exacerbated by the crowding-out caused by local government debt, which leads to stronger motives for leverage manipulation (e.g., Rao et al., 2022). Among them, listed firms may hide the true leverage by using convertible and mixed debt as firm equity and designing leasing operations as off-balance sheet liabilities (e.g., Scott et al., 2011; Callahan et al., 2012; Kraft, 2015).

In terms of the influence of external governance factors, Zhai et al. (2021) investigate from the perspective of party organization governance that party organizations can inhibit firm leverage manipulation by improving firm information transparency and reducing management’s opportunistic motives when leverage manipulation does exist in SOEs. Based on the perspective of institutional investors’ “distraction,” other studies have verified that institutional investors, who are “distracted” by the difference in attention allocation in the portfolio, are unable to play a good role in identifying and monitoring and that firms take advantage of the weak governance effect of institutional investors and the reduced information content of share prices to manipulate leverage (Wu et al., 2022).

However, existing studies consider the relationship with leverage manipulation from the “external factors” of the firm, lacking attention to the internal factors. Firm financing needs and leverage manipulation depend on operational and strategic alignment. Based on this, this study attempts to investigate the relationship between strategic differentiation and firm leverage manipulation using financing constraints as a mediator to enrich the literature on the factors influencing firm leverage manipulation and provide empirical support for preventing firms from a thirst quencher to short-term benefits and promoting the effective implementation of deleveraging policies.

### Institutional background

Since the international financial crisis outbreak, “reducing leverage and deleveraging” has become the basic consensus for major developed economies to get out of the crisis. While emerging economies do not have high-risk leverage ratios, and the urgency to deleverage is relatively low, China cannot afford to take this lightly. In response to the huge impact of the international financial crisis, China launched a massive economic stimulus package in 2008, and its macro leverage ratio has soared as a result. Prior studies (e.g., Zhang et al., 2018) indicate that the leverage ratio increased by 86.2% in just 7 years from 2008 to 2015, with an average annual growth rate of more than 12%.

The rapidly rising leverage ratio has drawn great attention from the Chinese central government. In this context, China has been focusing on promoting the deleveraging policy with the main purpose of preventing systemic financial risks since 2016. The Central Committee of the Communist Party of China pointed out one of the five major reform tasks for 2016 – deleveraging – when it held its 2015 annual economic work conference. General Secretary Xi also stressed at the 2017 National Financial Work Conference that the relationship between structural adjustment, stable growth, and total control should be handled to achieve economic deleveraging. In addition, the reduction of leverage of SOEs should be prioritized, and “zombie firms” should be disposed of.

After a year, the Central Finance and Economics Commission proposed for the first time to take structural deleveraging as the basic idea and targeted different requirements by sector and debt type, emphasizing efforts to achieve a steady reduction of the macro leverage ratio. As a result, the series of policy evolution can be summarized as “general deleveraging – firm deleveraging – SOE deleveraging becomes the focus – structural deleveraging,” which not only reflects the gradual deepening of policymakers’ understanding of the high leverage problem but also reveals the increasingly precise deleveraging measures and the government’s determination to deleverage.

From 1999 to 2016, China’s leverage took the lead after four times to reach a stage peak. At the same time, China experienced four times “deleveraging” in 1999, 2004, 2011, and 2016. These four stages of deleveraging occurred in different contexts, and the policies adopted differed.

In 1999, affected by the Asian financial crisis, China’s economic growth rate reached an all-time low, hindering the development of China’s major firms and raising the financial risks brought about by the rising leverage ratio. The Chinese Academy of Social Sciences shows that China’s leverage ratio peaked in 1999. From 1995 to 1999, the leverage ratio of the non-financial sector rose from 80.95% to 97.21%, and the leverage ratio of the residential sector rose from 8.24% to 12.24%. In addition, the leverage ratio of the government sector rose from 8.66% to 18.76%, while there were large losses of SOEs, overcapacity, and a vicious circle of debt and deflation.

To this end, China has launched a “deleveraging” process with the reform of SOEs as the core. Through the market-oriented reform policy of SOEs, the establishment of four major asset management firms, deleveraging for the four major state-owned banks, issuing additional treasury bonds to fully support effective demand, increasing the quantity of money supply to stimulate consumer demand, and many other initiatives to make effective demand pick up and expand, economic growth rebounded and deleveraging had significant effects.

Around 2004, China’s fixed asset investment soared, money supply growth accelerated, and the macro environment economic overheating background, the macro leverage ratio in various sectors climbed once again. According to the Chinese Academy of Social Sciences, the leverage ratio of the non-financial sector increased by nearly 13% during this period, only slightly lower than 17% in 1999. As a result, the Chinese government adopted a series of policies in the second quarter of 2004 to alleviate the high leverage triggered by the overheated economy. The demand structure has been reasonably improved, and the leverage ratio has been effectively reduced by raising the reserve deposit ratio, adjusting the refinancing rate and rediscount rate, increasing fiscal expenditures to services for people’s livelihood (special funding for the three rural areas, people’s employment, science, education, culture, and health), adjusting import and export tariffs and increasing reform and opening up, and carrying out a new round of tax reform to boost demand. In particular, the non-financial sector leverage ratio fell to 95.2% in 2008, down nearly 11% compared to 2004.

Leverage peaked around 2011 when inflationary pressures came to the fore in all sectors of the country. Statistics from the Chinese Academy of Social Sciences show that from 2008 to 2010, the leverage ratio of the non-financial sector soared from 95.2% to 120.34%, while the leverage ratio of the residential sector almost doubled. As a result, the government’s monetary policy has been steadily tightened, the cost of livelihood spending has been guaranteed, real estate regulation policies have been tightened, leverage has been effectively controlled, and inflation has been significantly reduced.

Compared with the previous three “deleveraging,” the government “deleveraging” in 2016 was much stronger. The central and local governments are not only pressing ahead with regulatory policies in multiple areas involving trust risks, bank wealth management, and management of securities investment fund subsidiaries but have also set up a special State Council Financial Stability Development Committee in terms of organizational structure. In addition, establishing the China Banking and Insurance Regulatory Commission in 2018 reflects even more, the importance attached to the sustainable development of the financial industry at the national level. Since 2016, China’s deleveraging policy has achieved initial results, with China’s macro leverage ratio decreasing by 1.5% in 2018 compared to an average annual increase of more than 10% before 2018 (Zhang and Wan, 2020).

### Hypothesis development

Prior studies (e.g., Zhang and Rajagopalan, 2010; Xu et al., 2021) have found an inverted U-shaped relationship between strategic differentiation and firm innovation and operational performance. Within a certain range, improving the strategic differentiation of a firm can lead to better technological innovation and higher performance. Different strategies may help firms gain a competitive advantage. However, increased strategic differentiation can lead to more stringent financing constraints in terms of both information risk and operational risk.

On the one hand, from the perspective of information risk, the degree of strategic differentiation has a negative impact on the “quantity” and “quality” of firm information available to capital markets. Strategic differentiation increases the cost of analysts’ access to information by increasing the degree of firm information asymmetry, leading to a reduction in the number of securities analysts to follow (He and Yin, 2018), further cutting down the effective information sources for investors and the amount of firm information available to external markets.

In addition, firms with higher strategic differentiation have more complex organizational structures and diverse operational activities, increasing the difficulty for stakeholders to judge the financial condition as well as the operating performance of firms based on industry common sense and experience and increasing the forecast error and forecast disagreement of financial analysts (Kochhar and Hitt, 1998; Huang et al., 2018; Hu and Zheng, 2021). Moreover, the rising cost of resource acquisition and increased operational risks force firms to reduce information transparency and even increase the tendency of firm disclosure irregularities (Wang and Li, 2020), which in turn reduces the quality of firm information obtained from external markets.

On the other hand, from the perspective of operational risk, strategic differentiation increases the cost and difficulty of resource reallocation, increases the magnitude of future cash flow volatility and loan default risk (Li and Yu, 2021), and generates more extreme operational performance (Gao and Chen, 2020), leading to an escalation of operational risk. As a result, investors at an information disadvantage are unable to make more accurate judgments about the real situation of the firm and, taking into account, the risk of operational operation or even bankruptcy. They demand a certain risk–reward or choose to set stricter restrictive terms and other means to guarantee their rights and interests (Wang et al., 2017; Huang et al., 2018; Hu and Zheng, 2021), which undoubtedly for firms that need more financing to achieve differentiation (Acharya et al., 2018). A firm’s leverage ratio is a basic indicator of its solvency and a measure of financial risk. Accordingly, external financial institutions pay great attention to the leverage position of firms in their credit decisions, intending to reduce credit risk. For this reason, driven by financing pressure, firm managers and shareholders have incentives to reduce firm leverage to enhance their financing capacity and satisfy their debt covenants (Xu et al., 2021).

However, the higher the degree of strategic differentiation, the higher the operational risk of the firm, which not only needs funds to maintain operations but also needs to maintain sufficient cash to deal with unexpected problems in the course of operations, making it more difficult to come up with spare funds to repay debts (Gao and Chen, 2020). The increased volatility of operating performance is more likely to lead to weakened sustainability of future cash flows and profitability of firms, increased possibility of capital chain breakage, and the inability of firms to ensure real deleveraging by increasing external equity financing or retained earnings (Ma and Yiu, 2021). While substantive deleveraging is difficult, artificially reducing firm financial leverage by manipulating leverage through off-balance sheet liabilities, nominal equity but real debt, and accounting manipulation can not only send a “good” signal to the investors at a relatively low cost but also increase firm financing capacity (Xu and Lu, 2020).

Therefore, in an environment where financing is difficult, and the government is under pressure to “deleverage,” management and shareholders of firms with high strategic differentiation have a strong incentive to choose leverage manipulation as a way to quench their thirst to formally comply with policies to obtain a more relaxed financing and regulatory environment. Accordingly, the first hypothesis is proposed in this study.

*H1*: The degree of strategic differentiation is positively associated with the likelihood that firms engage in leverage manipulation.

One of the main purposes of leverage manipulation by strategically differentiated firms is to increase their financing capacity, meet the higher financing needs needed to implement their differentiated strategies, signal to the investors that they are in a good financial position, and reduce operational stress. However, extant studies demonstrate that the contradiction between the growing financing needs of firms and the increasingly stringent financing constraints is highlighted in the context of increased strategic differentiation (Luo and Tian, 2018). Short-term debt repayment pressure seriously affects a firm’s ability to raise funds. The relative lack of fundraising ability prevents the firm from obtaining funds to meet investment and production operations, which in turn affects the growth of sales and profits and poses a serious challenge to the long-term sustainable development of the firm. Therefore, when the short-term debt repayment pressure is higher, differentiated firms, which already need to maintain sufficient cash to cope with risks, rely more on financial institutions to obtain the required funds and have more incentives to reduce their leverage levels using nominal equity and real debt including off-balance sheet liabilities, convertible bonds, and structured principal investments (e.g., Feng et al., 2009; Scott et al., 2011) to alleviate their short-term debt repayment pressure. As a result, firms with higher short-term debt repayment pressure have stronger incentives to manipulate leverage. Accordingly, the second hypothesis is proposed in this study.

*H2*: The association between strategic differentiation and leverage manipulation is more significant in firms with high short-term debt repayment pressure.

Industry product market competition can influence the country’s industrial structure on a macro level and the firm’s operational decisions on a micro level. For example, Lai (2012) finds that competition in the market can promote the resource allocation efficiency of firms, while Fang (2011) points out that intense market competition can weaken the industry’s returns. Therefore, as an important external factor, industry competition is bound to impact operational decisions.

An important issue is how the degree of market competition affects the relationship between strategic differentiation and firm leverage manipulation. In terms of financing efficiency, when the market is competitive, the firm’s risk of bankruptcy increases, and banks and other financial institutions reasonably expect “competitive risk” and reduce their financial support to the firm and adjust the size of the loan interest rate accordingly (Paul and Michael, 2004; Chen et al. 2005; Feng, 2021; Lin and Tang, 2021). Chen et al. (2012) analyze that market competition affects firms’ profitability mainly by lowering market prices and increasing cash flow volatility, making firms face more serious financing constraints.

In addition, Han and Zhou (2011) find that when firms are in a more competitive market environment, their need to reserve funds through debt to cope with risk is enhanced. In other words, in the case of higher strategic differentiation, the firms’ financing needs have increased while the difficulty of financing has risen. The competition in the industry product market has increased the firms’ capital storage needs and intensified the financing constraints.

In terms of profitability level, the increased degree of competition in the industry’s product market can cause firms to face the problems of small product differentiation, high financing constraints, and low customer stickiness and to take up market share. As a result, firms often need to adopt price wars and marketing wars, resulting in lower excess profits (Zhou and Zhou, 2014; Jiang and Xing, 2021). Moreover, Zhou and Wang (2017) also find that the buyer’s market is advantaged in a fiercely competitive industry environment, intensifying firms’ financial and operational risks.

In addition, Yao et al. (2018) find that in a competitive environment, less competitive and leading firms choose to disclose less useful information to ensure the firm’s dominant position in the competitive market for their products. Finally, Yuan et al. (2017) find that in terms of the proprietary cost effect, firms’ willingness to disclose financial information decreases as the degree of competition increases, making it more difficult for analysts to gather information, exacerbating information asymmetry, and reducing the comparability of accounting information. Therefore, in the competitive environment of the industry product market, the greater degree of financing constraints and higher operational risks intensify the positive effect of strategic differentiation on firm leverage manipulation, and the higher information opacity increases leverage manipulation. Accordingly, the third hypothesis is proposed in this study.

*H3*: The higher the degree of product market competition in a firm’s industry, the more significant the positive relationship between strategic differentiation and leverage manipulation.

External audit plays a governance role by improving the quality of audited financial reports, mitigating agency problems, and reducing information asymmetry, which is an important part of corporate governance. On the other hand, auditor industry expertise reflects the auditor’s professional competence in a specific industry or field. Therefore, auditors with industry expertise are better able to design and execute audit procedures, collect evidence, and improve the quality of audit reports based on the operation characteristics, industry expertise, transaction processes, and related accounting policies and methods of the audited entity’s industry (Song et al., 2016). As a result, auditors with industry expertise can better exercise supervisory control over firms’ more insidious leverage manipulation.

On the one hand, in terms of auditors’ expertise, auditors with industry expertise can indirectly weaken the possibility of firm leverage manipulation by reducing firm financing costs and directly discourage firm leverage manipulation through a supervisory role. First, auditors with industry expertise can enhance investors’ confidence and reduce financing constraints for firms. Krishnan et al. (2013) find that auditors with industry expertise can reduce the information risk of stakeholders by improving the quality of the audited entity’s accounting information, which in turn reduces the return on risk required by investors and achieves the effect of reducing the firm’s cost of equity capital. Chang et al. (2016) also point out that auditors with industry expertise can reduce information asymmetry, reduce agency costs, enhance creditors’ investment confidence, mitigate information premium risk, and reduce debt financing costs.

Second, auditors with industry expertise can improve the quality of their audits, timely detection of irregularities and violations of the audited entity, and play a direct monitoring and control role. As a result, industry audit experts with extensive experience can provide higher audit quality (Moroney, 2007; Gaver and Utke, 2019) and inhibit earnings management (e.g., Balsam et al., 2000; Krishnan, 2003; Kwon et al., 2007; Zhang et al., 2012), and reduce the probability of financial restatement in the audited entity (e.g., Jayaraman and Milbourn, 2015), which contributes to the improvement of audit efficiency and financial reporting quality. For example, the Ministry of Finance’s Accounting Standard for Business Enterprises No. 6 – Intangible Assets, issued in 2006, provides that a firm’s R&D expenditures can be conditionally capitalized. However, capitalization terms are heavily influenced by management subjectivity. Capitalization of R&D expenditures has become a new means of accounting manipulation by management (e.g., Gang and Zhu, 2010). Auditor industry expertise can inhibit such manipulative behavior of firms (e.g., Chu, 2018). Chen et al. (2010) also find that contingency accounting and the manager’s information advantage provide opportunities for firms to perform manipulations. They are more likely to conceal information on contingencies such as material debt guarantees and pending litigation. Auditors with industry expertise can better identify and correct such behaviors by management and improve the quantity and quality of disclosure of contingent information.

On the other hand, in terms of the auditor’s motivation to provide high-quality audit services, auditors with industry experts take the initiative to exercise their expertise to more accurately and objectively evaluate the fairness of their client’s financial reports, which can be divided into the following three aspects. First, auditors with industry experts take the initiative to exercise their expertise to protect the industry’s reputation. Based on the reputation theory, auditors with industry expertise can respond proactively after identifying firm violations and eliminate opportunistic behaviors such as management information manipulation and concealment of bad news (e.g., Niu and Accounting, 2013). Second, auditors with industry experts perform more disciplined audits to reduce the firm’s investment losses. Industry expertise stems from ongoing investments in the specific industry in which the firm operates. Hence, the more specialized investments in people, technical methods, and internal management controls that auditing firms with industry expertise make after low-quality audits are exposed. As a result, the more investment losses the auditing firm bears (e.g., Zhang et al., 2012).

Third, auditors with industry expertise proactively urge firms to correct or disclose their reports to the public to reduce the likelihood of litigation damages. According to the “deep pocket theory,” auditing firms with industry expertise usually also have the capacity for civil damages. Therefore, the likelihood of an auditing firm being ordered to pay damages in the event of a failed audit due to undetected misstatements increases significantly (Chen et al., 2010). Thus, auditors with industry experts have a greater incentive to detect and reveal financial statements misstatements to maintain their reputation and auditing firm brand and avoid the risk of litigation damages, thereby discouraging firm leverage manipulation. Accordingly, this study proposes the fourth hypothesis.

*H4*: Auditors with industry expertise weaken the positive relationship between strategic differentiation and firm leverage manipulation.

## Research design

### Sample selection and data sources

This study utilizes the A-share listed firms in Shanghai and Shenzhen from 2007 to 2020 as the research sample. This study then makes the following deletions: (1) firms in the finance and insurance industries due to their special characteristics; (2) the ST and *ST firms; (3) other samples with missing data. Finally, this study obtains 19,773 firm-year observations. To reduce the effect of extreme values of variables on the findings of the study, the continuous variables used are winsorized at both the 1% and 99% levels. All other data used in this study are obtained from the CSMAR system, China’s leading financial data service firm.

### Variable definition

Xu et al. (2020) demonstrate that firms can manipulate leverage through off-balance-sheet liabilities, nominal equity but real debt, and accounting manipulation and propose an innovative measure of leverage manipulation that can provide a more comprehensive and integrated measure of the extent of firm leverage manipulation. Considering that the basic XLT-LEVM method is more scientifically calculated, this study uses the leverage manipulation measured by the basic XLT-LEVM method to measure the dependent variable in the main test. In addition, this study performs robustness tests using the leverage manipulation measured by the extended XLT-LEVM method. Specifically, following Xu et al. (2020) and Xu et al. (2021), the basic XLT-LEVM method to calculate leverage manipulation is formulated as follows.


(1) $$\begin{array}{l} {\text{LEVM}}_{i,t}=\left(\begin{array}{l} \text{DEBTB}\_{\text{TOTAL}}_{i,t}+\text{DEBT}\_OB_{i,t}\\ +\text{DEBT}\_NSRD_{i,t} \end{array}\right)\\ \;/\left(\text{ASSET}\_\text{TOTA}L_{i,t}+\text{DEBT}\_OB_{i,t}\right)-\text{LEV}B_{i,t} \end{array}$$

Where ${\text{LEVM}}_{I,t}$ is the firm leverage manipulation. DEBTB_TOTAL*_i,t_* is total liabilities. *ASSET*_*TOTAL_i,t_* is total assets. $\text{LEV}B_{i,t}$ is the firm leverage. *DEBT*_*OB_i,t_* and $\text{DEBT}\_NSRD_{i,t}$ are the sum of off-balance sheet liabilities and nominal equity but real debt estimated by the expectation model method. The principle of estimation by the expectation model method is to construct a model using some available indicators of the firm, regress to measure the true expected value, and compare it with the firm’s book value. The book value greater than the expected value indicates that there is an off-balance sheet liability or nominal equity but real debt. The difference between the true expected value and the book value is the sum of off-balance sheet liabilities or nominal debt but real debt. The book value less than the expected value denotes that there is no outlier.

Moreover, following Tang et al. (2011), Gao and Chen (2020), and John and John (2007), this study uses the following six-dimensional indicators to measure strategic differentiation: (1) capital intensity (fixed assets/number of employees); (2) advertising and promotion investment (advertising expenses/sales revenue); (3) R&D intensity (R&D expenditure/sales revenue); (4) overhead efficiency (overhead/sales revenue); (5) fixed asset renewal degree (net fixed assets/original value of fixed assets); and (6) financial leverage (total liability/owner’s equity). First, since the firms in China do not disclose advertising expenses and there has been less disclosure of R&D expenditures in previous years, this study uses selling expenses and net intangible assets instead. Second, the six indicators are standardized. Specifically, the deviation of each indicator from the industry average is obtained by subtracting the average value of each industry for each of the six indicators, dividing by the standard deviation of each industry, and taking the absolute value. Finally, the deviations of these six indicators are weighted and averaged to obtain the firm’s strategic differentiation (SD). The higher the value of this indicator, the greater the deviation from the average level of strategy of the firm and the firm in its industry.

Furthermore, this study employs firm size (SIZE), profitability level (ROA), firm cash flow (CFO), firm growth (GROWTH), the gearing ratio (LEVB), market-to-book ratio (TOBIN), percentage of independent directors (INDEPENDENT), and board size (BOARD) as control variables (e.g., Xu et al., 2021; Zhai et al., 2021). The specific variable names and definitions are shown in Table 1.

**Table 1:** Variable definition.

Names	Symbols	Variable definitions
Leverage manipulation	LEVM	Basic XLT-LEVM Method Measurements
Strategic differentiation	SD	The average deviation from the industry average for six strategic dimensional indicators
Short-term debt repayment pressure	PRESS	(current liabilities + non-current liabilities due within 1 year) − net cash flow from operating activities and divided into two groups according to the industry annual median, with the higher group taking 1 and 0 otherwise
Auditors industry expertise	IMS	The ratio of the client’s total audit fees to the total audit fees of all listed firms in the client’s industry, with 10% as the threshold and 1 for deciles above; otherwise, 0
Firm size	SIZE	Natural logarithm of total firm assets
Profitability level	ROA	Net profit/total assets
Firm cash flow	CFO	Cash flows from operating activities
Firm growth	GROWTH	(Current amount of operating income for the current year – amount for the same period of the previous year)/(Amount of operating income for the same period of the previous year)
Gearing ratio	LEVB	Financial leverage of the firm
Market-to-book ratio	TOBIN	Book value/Market value
Percentage of independent Directors	INDEPENDENT	The ratio of independent directors to the number of board of directors
Board size	BOARD	Total number of board of directors

### Model design

To test the research hypotheses of this study, the following model is constructed.


(2) $$\begin{array}{l} \text{LEV}M_{i,t}=\alpha_{0}+\alpha_{1}SD_{i,t}+\alpha_{2}\sum CV_{S}\\ \;+\sum \text{Industry}+\sum \text{Year}+\varepsilon_{i,t} \end{array}$$

Where CVs is the control variable, including firm size (SIZE), profitability level (ROA), firm cash flow (CFO), firm growth (GROWTH), the gearing ratio (LEVB), market-to-book ratio (TOBIN), percentage of independent directors (INDEPENDENT), board size (BOARD) and equity concentration (FIRST). In addition, industry effects and year effects are controlled for in the model with heteroskedasticity robust standard errors.

### Descriptive statistics

Table 2 shows the results of descriptive statistics for the main variables. The results show that the mean value of firm leverage manipulation under the basic XLT-LEVM method measure is 0.114, indicating the extent of leverage manipulation through off-balance-sheet liabilities and nominal equity but real debt is about 11.4% for listed firms in this study. The mean value of leverage manipulation under the extended method (direct method) measure is 0.117, indicating the extent to which firms manipulate leverage through off-balance sheet liabilities, nominal equity but real debt and depreciation of fixed assets, and capitalization of R&D expenditures is 11.7%. Consequently, listed firms are often leveraged through off-balance-sheet liabilities and nominal equity but real debt. The mean value of the strategic differentiation is 0.664. The standard deviation of the strategic differentiation is 0.319. The maximum value of the strategic differentiation is 2.214. The minimum value of the strategic differentiation is 0.195. The results show that there is a large variation in the strategic differentiation of the firms in this study.

**Table 2:** Descriptive statistics of main variables.

Variables	(1)	(2)	(3)	(4)	(5)	(6)	(7)	(8)
	*N*	Mean	SD	p25	med	p75	min	max
LEVM	19,773	0.114	0.184	0.000	0.046	0.164	0.000	1.225
ExpLEVM	19,773	0.117	0.184	0.002	0.048	0.165	0.000	1.232
ExpLEVMI	19,773	0.115	0.189	0.005	0.050	0.165	−0.093	1.251
SD	19,773	0.664	0.319	0.445	0.589	0.803	0.195	2.124
LEVB	19,773	0.438	0.194	0.288	0.429	0.578	0.043	2.627
SIZE	19,773	22.235	1.273	21.317	22.046	22.943	18.367	26.430
CFO	19,773	0.047	0.065	0.010	0.045	0.085	−0.251	0.327
ROA	19,773	0.038	0.074	0.014	0.039	0.072	−0.588	0.257
GROWTH	19,773	0.175	0.434	−0.016	0.111	0.266	−0.847	7.619
TOBIN	19,773	2.006	1.236	1.244	1.615	2.298	0.811	11.443
INDEPENDENT	19,773	0.375	0.054	0.333	0.333	0.429	0.250	0.600
BOARD	19,773	2.136	0.197	1.946	2.197	2.197	1.609	2.708

All variables as previously defined.

### Coefficient correlation test

The results of Pearson correlation coefficients are presented in Table 3. Table 3 shows that the firm strategic differentiation is significantly and positively associated with leverage manipulation. The result preliminarily indicates that the greater the firm strategic differentiation, the higher the likelihood of leverage manipulation. After model regression, the maximum value of the variance expansion factor (VIF) was 2.11, both below the 10-warning criterion (Kennedy, 1998), indicating that there was no significant Multicollinearity between the variables.

**Table 3:** Coefficient correlation test.

Variables	LEVM	SD	SIZE	ROA	CFO	LEVB	TOBIN	INDEPENDENT	BOARD
LEVM	1.000								
SD	0.027***	1.000							
SIZE	−0.026***	0.018**	1.000						
ROA	0.044***	−0.200***	0.043***	1.000					
CFO	0.026***	−0.063***	0.093***	0.356***	1.000				
LEVB	0.063***	0.140***	0.458***	−0.343***	−0.141***	1.000			
TOBIN	0.009	0.035***	−0.402***	0.138***	0.088***	−0.278***	1.000		
INDEPENDENT	−0.009	0.032***	0.048***	−0.021***	−0.010	−0.002	0.032***	1.000	
BOARD	0.005	−0.011	0.237***	0.036***	0.037***	0.139***	−0.125***	−0.504***	1.000

***, **, * denote 1%, 5%, and 10% significance levels, respectively. All variables as previously defined.

## Empirical results and analysis

### Strategic differentiation and leverage manipulation

Table 4 reports the regression results for the association between strategic differentiation and firm leverage manipulation. Column (1) shows the results without controlling the effect of industry and year. Columns (2), (3), and (4) show the results of controlling for industry effects, year effects, and simultaneous industry and year effects, respectively. In columns (1)–(4), the regression coefficients of strategic differentiation (SD) are consistently positive and significant at the 1% level, indicating that for firms with greater strategic differentiation, leverage manipulation through off-balance sheet liabilities and nominal equity but real debt is more likely, supporting the H1. In terms of control variables, gearing is positively associated with leverage manipulation, which suggests that highly leveraged firms are more likely to leverage manipulation, in line with the findings of existing studies (e.g., Landsman et al., 2008; Scott et al., 2011; Christensen and Nikolaev, 2013; Kraft, 2015).

**Table 4:** Strategic differentiation and firm leverage manipulation.

	(1)	(2)	(3)	(4)
	LEVM	LEVM	LEVM	LEVM
SD	0.017***	0.014***	0.018***	0.016***
	(3.718)	(3.032)	(3.972)	(3.405)
SIZE	−0.014***	−0.017***	−0.016***	−0.020***
	(−10.170)	(−12.281)	(−11.105)	(−13.446)
ROA	0.215***	0.244***	0.232***	0.265***
	(7.866)	(8.928)	(8.224)	(9.381)
CFO	0.067***	0.037	0.069***	0.035
	(2.715)	(1.511)	(2.688)	(1.375)
GROWTH	0.002	0.002	0.002	0.002
	(0.428)	(0.476)	(0.425)	(0.533)
LEVB	0.131***	0.131***	0.138***	0.139***
	(12.818)	(12.163)	(13.089)	(12.581)
TOBIN	−0.002**	−0.002	−0.005***	−0.004***
	(−2.075)	(−1.357)	(−3.646)	(−3.078)
INDEPENDENT	−0.005	−0.001	0.002	0.008
	(−0.201)	(−0.027)	(0.063)	(0.306)
BOARD	−0.005	−0.003	0.001	0.005
	(−0.618)	(−0.369)	(0.070)	(0.517)
_cons	0.363***	0.395***	0.407***	0.450***
	(10.579)	(10.212)	(10.588)	(10.601)
*N*	19,773	19,773	19,773	19,773
*R*^2^_a	0.016	0.065	0.018	0.067
Industry	NO	YES	NO	YES
Year	NO	NO	YES	YES

Values in parentheses are *t*-statistics; ***, **, * denote 1%, 5%, and 10% significance levels, respectively. All variables as previously defined.

### Heterogeneity analysis of the effect of short-term debt repayment pressure on leverage manipulation

Following Xu et al. (2021), which measures the short-term debt repayment pressure of firms by the sum of current liabilities and non-current liabilities due within 1 year minus net cash flow from operating activities, this study divides the sample into two groups with low short-term debt repayment pressure and high short-term debt repayment pressure by the median. This study then uses the suest (test based on the seemingly uncorrelated model SUR) method to test for differences in coefficients between groups. The regression results are shown in Table 5.

**Table 5:** Strategic differentiation and firm leverage manipulation.

	LEVM	
	(1) PRESS = 0	(2) PRESS = 1
SD	−0.007	0.037***
	(−1.160)	(6.417)
SIZE	−0.007**	−0.024***
	(−2.501)	(−12.315)
ROA	0.301***	0.226***
	(9.898)	(7.538)
CFO	0.038	−0.001
	(1.251)	(−0.042)
GROWTH	−0.008*	0.010**
	(−1.714)	(2.315)
LEVB	0.144***	0.139***
	(11.857)	(10.818)
TOBIN	−0.001	−0.005**
	(−0.404)	(−2.446)
INDEPENDENT	−0.013	0.042
	(−0.317)	(1.095)
BOARD	0.001	0.006
	(0.084)	(0.524)
_cons	0.187**	0.538***
	(2.539)	(8.561)
*N*	9,889	9,884
R^2^_a	0.067	0.087
Industry	YES	YES
Year	YES	YES
Suest test for difference in coefficients between groups:	*χ*^2^(1) = 23.290 Prob > *χ*^2^ = 0.000	

Values in parentheses are *t*-statistics; ***, **, * denote 1%, 5%, and 10% significance levels, respectively. All variables as previously defined.

Column (1) is the group with lower short-term debt repayment pressure. Column (2) is the group with higher short-term debt repayment pressure. The strategic differentiation (SD) regression coefficient for the group with higher short-term debt repayment pressure is 0.037 and is significant at the 1% level. The group’s strategic differentiation regression coefficient with lower short-term debt repayment pressure is negative and insignificant. The difference between the two groups of regression coefficients passed the significance test, indicating that short-term debt repayment pressure increases the likelihood of leverage manipulation by firms with greater strategic differentiation, supporting the H2.

### Heterogeneity analysis of the effect of industry product market competition on leverage manipulation

Based on Zhou and Zhou (2014) and Liu et al. (2003), this study measures the degree of industry product market competition of firms using the number of firms in the industry to which they belong and the share of sales revenue of the top four firms in the industry, respectively, and divides the sample into two groups of low and high industry product market competition through median grouping, and uses the suest (test based on the seemingly uncorrelated model SUR) method to test for differences in coefficients between groups (Lian and Liao, 2017). The regression results are shown in Table 6.

**Table 6:** Industry product market competition, strategic differentiation, and firm leverage manipulation.

	LEVM			
	(1) competition1 = 0	(2) competition1 = 1	(3) competition2 = 0	(4) competition2 = 1
SD	0.001	0.027***	0.006	0.024***
	(0.219)	(5.285)	(0.957)	(4.430)
SIZE	−0.019***	−0.020***	−0.016***	−0.024***
	(−8.799)	(−10.669)	(−7.962)	(−11.516)
ROA	0.190***	0.332***	0.195***	0.319***
	(5.740)	(12.187)	(6.051)	(11.222)
CFO	−0.046	0.128***	−0.041	0.125***
	(−1.403)	(4.467)	(−1.334)	(4.043)
GROWTH	0.008*	−0.005	0.002	0.003
	(1.847)	(−1.247)	(0.492)	(0.573)
LEVB	0.093***	0.180***	0.095***	0.182***
	(6.870)	(16.486)	(7.509)	(15.417)
TOBIN	−0.001	−0.006***	0.000	−0.007***
	(−0.535)	(−3.847)	(0.039)	(−4.116)
INDEPENDENT	−0.036	0.061	0.021	−0.012
	(−0.850)	(1.637)	(0.514)	(−0.298)
BOARD	0.010	−0.001	0.014	−0.008
	(0.803)	(−0.076)	(1.144)	(−0.674)
_cons	0.448***	0.391***	0.354***	0.588***
	(7.013)	(7.548)	(6.190)	(8.228)
*N*	9,776	9,997	9,798	9,975
*R*^2^_a	0.074	0.055	0.077	0.059
Industry	YES	YES	YES	YES
Year	YES	YES	YES	YES
Suest test for difference in coefficients between groups:	*χ*^2^(1) = 7.400 Prob > *χ*^2^ = 0.0065		*χ*^2^(1) = 3.840 Prob > *χ*^2^ = 0.0500	

Values in parentheses are *t*-statistics; ***, **, * denote 1%, 5%, and 10% significance levels, respectively. All variables as previously defined.

Columns (1) and (2) show the regression results under the measure of the number of firms in the industry to which the firm belongs. In column (2), where the industry product market competition is high, the regression coefficient for the association between strategic differentiation and firm leverage manipulation is significantly positive at the 1% level with a regression coefficient of 0.027. Columns (3) and (4) show the regression results under the measure of the share of sales revenue of the top four firms in the industry. The results are consistent with those under the measure of the number of firms in the industry to which the firm belongs, with a regression coefficient of 0.024 for the more competitive group, which is significantly positive at the 1% level. The results indicate that the higher the degree of strategic differentiation, the higher the possibility of firm leverage manipulation when the industry product market competition is high, supporting the H3 of this study.

### Heterogeneity analysis of the impact of auditor industry expertise on leverage manipulation

Existing studies commonly use proxy variables for auditors’ industry expertise: the industry market share method and the industry portfolio share method. The industry market share method is more common in the Chinese audit market. This study follows Wang et al. (2020) to measure auditor industry expertise. The ratio of the total audit fees of the firm to the total audit fees of all listed firms in the firm’s industry is first calculated as IMS. Then, 10% is used as the threshold to classify the auditor’s industry expertise. If IMS is above the 10th percentile, the auditor’s industry expertise variable takes 1. Otherwise, it is 0. Next, the groups are grouped by IMS dummy variables and tested for differences in coefficients between groups using the suest method. The regression results are shown in Table 7.

**Table 7:** Auditors’ industry expertise, strategic differentiation, and firm leverage manipulation.

	LEVM	
	(1) IMS = 0	(2) IMS = 1
SD	0.019***	−0.017
	(4.465)	(−1.043)
SIZE	−0.021***	−0.013**
	(−13.097)	(−2.514)
ROA	0.285***	0.061
	(12.861)	(0.761)
CFO	0.034	0.071
	(1.478)	(0.986)
GROWTH	0.002	0.007
	(0.603)	(0.751)
LEVB	0.139***	0.124***
	(15.404)	(3.936)
TOBIN	−0.005***	0.013**
	(−4.005)	(2.444)
INDEPENDENT	0.020	−0.138
	(0.652)	(−1.484)
BOARD	0.003	−0.007
	(0.374)	(−0.260)
_cons	0.471***	0.373***
	(9.963)	(3.115)
*N*	17,759	2014
*R*^2^_a	0.068	0.082
Industry	YES	YES
Year	YES	YES
Suest test for difference in coefficients between groups:	*χ*^2^(1) = 5.58 Prob > *χ*^2^ = 0.0182	

Values in parentheses are *t*-statistics; ***, **, * denote 1%, 5%, and 10% significance levels, respectively. All variables as previously defined.

Column (1) shows the regression results for auditors who do not have industry expertise. The regression coefficient of strategic differentiation in this group is significantly positive at the 1% level with a regression coefficient of 0.019. Column (2) shows the regression results for auditors with industry expertise. The regression coefficient of strategic differentiation is negative and insignificant. The results indicate that auditors’ industry expertise can weaken the positive relationship between strategic differentiation and firm leverage manipulation, which supports the H3 of this study.

## Possible mechanism effect

### Financing constraint mechanism

The theoretical analysis mentioned above shows that strategic differentiation enhances the likelihood of leverage manipulation by increasing a firm’s financing constraints. To further verify whether the transmission path of “strategic differentiation – financing constraint – firm leverage manipulation” holds, this study follows previous studies (e.g., Whited and Wu, 2006; Dmitry et al., 2009) to measure the financing constraint of firms using the WW index, and empirically investigates it using a stepwise test through a mediating effects model. Column (2) of Table 8 shows that strategic differentiation is positively related to firms’ financing constraints at the 1% level, i.e., the strategic differentiation exacerbates the difficulty of obtaining financing. The result is consistent with the existing studies (e.g., Wang et al., 2017; Hu and Zheng, 2021). The regression results in column (3) show that after adding the mediating variable (WW) to the model, the strategic differentiation and financing constraints are significantly and positively related to firm leverage manipulation at the 1% level. The results suggest that financing constraints partially mediate between strategic differentiation and leverage manipulation, i.e., strategic differentiation increases the likelihood of firm leverage manipulation by increasing firms’ financing constraints, which is consistent with the theoretical analysis mentioned above.

**Table 8:** Test of financing constraint mechanism.

	(1)	(2)	(3)
	LEVM	WW	LEVM
SD	0.017***	0.005***	0.017***
	(3.436)	(2.730)	(3.429)
WW			0.007**
			(2.228)
SIZE	−0.020***	−0.052***	−0.020***
	(−12.413)	(−19.126)	(−12.113)
ROA	0.307***	−0.024	0.307***
	(9.593)	(−0.448)	(9.598)
CFO	0.032	−0.143***	0.033
	(1.143)	(−10.358)	(1.179)
GROWTH	0.003	−0.123***	0.004
	(0.650)	(−4.050)	(0.821)
LEVB	0.143***	0.026***	0.143***
	(11.924)	(4.871)	(11.907)
FIRST	−0.023**	−0.022***	−0.023**
	(−2.290)	(−4.264)	(−2.275)
TOBIN	−0.004***	0.002***	−0.004***
	(−2.679)	(2.650)	(−2.689)
INDEPENDENT	0.015	0.011	0.015
	(0.516)	(0.379)	(0.513)
BOARD	0.009	0.010*	0.009
	(1.003)	(1.811)	(0.996)
_cons	0.448***	0.164***	0.447***
	(9.915)	(3.328)	(9.889)
*N*	17,086	17,086	17,086
*R*^2^_a	0.065	0.158	0.065
Industry	YES	YES	YES
Year	YES	YES	YES

Values in parentheses are *t*-statistics; ***, **, * denote 1%, 5%, and 10% significance levels, respectively. All variables as previously defined.

### Risk-taking level mechanism

Firms that choose higher levels of strategic differentiation usually need to take higher risks. Firms that have the drive and ability to actively choose a strategic model that deviates to a higher degree from the industry norm usually imply a higher level of acceptance of uncertainty, a more aggressive attitude toward risk, a more optimistic judgment about the apparent deleveraging that essentially increases the financial risk of the firm, and a greater likelihood of leverage manipulation. Wang et al. (2015) conclude that the more risk-averse people are, the more likely they are to invest in financial assets such as stocks, bonds, and funds. These derivatives have more complex, high-risk, and highly leveraged characteristics than typical financial assets. Yuan et al. (2019) also find that firms with higher strategic differentiation are more likely to engage in aggressive firm tax avoidance through a risk-taking level mechanism (Table 9).

**Table 9:** Risk taking level mechanism test.

	(1)	(2)	(3)
	LEVM	RISK	LEVM
SD	0.016***	0.013***	0.011*
	(3.336)	(6.586)	(1.866)
RISK1			0.089**
			(2.333)
SIZE	−0.020***	−0.002***	−0.023***
	(−13.162)	(−3.258)	(−12.028)
ROA	0.271***	−0.136***	0.464***
	(9.317)	(−9.757)	(11.345)
CFO	0.032	−0.008	−0.013
	(1.246)	(−0.933)	(−0.415)
GROWTH	0.002	0.007***	−0.000
	(0.424)	(4.699)	(−0.069)
LEVB	0.140***	−0.004	0.156***
	(12.598)	(−0.967)	(12.538)
TOBIN	−0.004***	0.002***	−0.005***
	(−2.768)	(3.719)	(−3.213)
INDEPENDENT	0.006	−0.022**	0.027
	(0.215)	(−2.269)	(0.823)
BOARD	0.004	−0.006**	0.013
	(0.414)	(−2.237)	(1.262)
_cons	0.446***	0.137***	0.448***
	(10.446)	(7.379)	(8.635)
*N*	19,472	13,054	13,054
*R*^2^_a	0.067	0.160	0.077
Industry	YES	YES	YES
Year	YES	YES	YES

Values in parentheses are *t*-statistics; ***, **, * denote 1%, 5%, and 10% significance levels, respectively. All variables as previously defined.

Therefore, the level of risk-taking may be a channel through which the degree of strategic differentiation affects firms’ leverage manipulation. Based on the method of Yuan et al. (2019), this study uses the industry-adjusted 3-year volatility of “EBITDA/total assets” of listed firms as a proxy for the level of firm risk-taking and the results of testing this path using a three-step approach are shown in Table 9. The degree of strategic differentiation and risk-taking in column (2) are significant at the 1% level. The regression results in column (3) show that strategic differentiation, risk-taking, and leverage manipulation are significantly positively correlated at the 1% level after adding the mediating variable (RISK) to the model. The results verify that risk-taking partially mediates between strategic differentiation and leverage manipulation. Therefore, the level of risk-taking is a channel through which strategic differentiation affects leverage manipulation.

## Robustness test

### Instrumental variables method

The previous study suggests that strategic differentiation increases the likelihood of firm leverage manipulation. Still, there may be endogeneity issues between strategic differentiation and leverage manipulation, in addition to the fact that there are more factors affecting leverage manipulation that are inevitably overlooked. To this end, this study adopts an instrumental variables approach to address the possible endogeneity problem based on Hu and Zheng (2021) and Li and Shi (2016), respectively.

Columns (1), (2), (3), and (4) of Table 10 present the results of the 2sls test with “lagged term of strategic differentiation” and “top five suppliers” as instrumental variables, respectively. The regression coefficient of strategic differentiation on leverage manipulation remains significantly positive at the 1% level in both instrumental variables tests. Both instrumental variables passed the Hausman test (*χ*^2^ = 193.700, *p* = 0.000; *χ*^2^ = 253.890, *p* = 0.000) as well as the weak instrumental variable test (*F* = 7839.270, *p* = 0.000; *F* = 211.521, *p* = 0.000).

**Table 10:** Instrumental variable method test.

Variables	(1)	(2)	(3)	(4)
	Stage1	Stage2	Stage1	Stage2
	SD	LEVM	SD	LEVM
LSD	0.746***			
	(135.53)			
Top 5 Supplier concentration			0.002***	
			(15.75)	
SD		0.023***		0.337***
		(3.89)		(7.93)
SIZE	0.011***	−0.021***	0.023***	−0.027***
	(5.91)	(−13.68)	(8.35)	(−13.97)
ROA	−0.450***	0.233***	−0.774***	0.498***
	(−16.52)	(10.27)	(−20.11)	(11.76)
CFO	−0.015	0.078***	−0.082*	0.072**
	(−0.52)	(3.23)	(−1.95)	(2.53)
GROWTH	−0.059***	0.006	−0.017***	0.001
	(−13.31)	(1.61)	(−2.79)	(0.25)
LEVB	0.064***	0.134***	0.111***	0.119***
	(5.53)	(13.99)	(6.69)	(10.04)
TOBIN	0.012***	−0.004***	0.029***	−0.014***
	(6.74)	(−3.03)	(12.36)	(−6.71)
INDEPENDENT	−0.047	0.014	0.108**	−0.032
	(−1.25)	(0.45)	(1.98)	(−0.88)
BOARD	−0.037***	0.009	−0.034**	0.013
	(−3.43)	(1.04)	(−2.13)	(1.18)
Constant	−0.017	0.467***	0.227***	0.346***
	(−0.33)	(11.00)	(2.79)	(6.07)
Year/Industry	YES	YES	YES	YES
Observations	15,256	15,256	16,192	16,192
*R*^2^_a	0.590	0.083	0.098	/

Values in parentheses are *t*-statistics; ***, **, * denote 1%, 5%, and 10% significance levels, respectively. All variables as previously defined.

Overall, the selection of instrumental variables is more reasonable. Therefore, after controlling for the endogeneity problem, the association between strategic differentiation and leverage manipulation is still significantly positive at the 1% level, indicating that the results of this study are robust.

### Firm fixed effects model

To eliminate the effects of unobserved variables that do not vary over time but do vary with individuals, following Xu et al. (2021), this study utilizes various measures of strategic differentiation and leverage manipulation to test the robustness of the main regression model using OLS firm fixed effects. The regression results are shown in Table 11. The regression coefficient of strategic variability is significantly positive at the 1% level, indicating that the results of this study are robust.

**Table 11:** Fixed effects test.

	(1)	(2)	(3)	(4)
	LEVM	ExpLEVMI	ExpLEVM	LEVM
SD	0.031***	0.028***	0.033***	
	(4.270)	(3.923)	(4.497)	
				
SD2				0.032***
				(5.204)
SIZE	−0.046***	−0.043***	−0.046***	−0.045***
	(−8.265)	(−7.890)	(−8.212)	(−8.209)
ROA	0.154***	0.511***	0.156***	0.154***
	(4.902)	(15.985)	(4.961)	(4.915)
CFO	−0.020	−0.489***	−0.022	−0.018
	(−0.681)	(−15.726)	(−0.747)	(−0.597)
GROWTH	0.012***	0.009*	0.012***	0.012**
	(2.613)	(1.912)	(2.616)	(2.566)
LEVB	0.081***	0.068***	0.085***	0.076***
	(3.631)	(3.308)	(3.834)	(3.448)
TOBIN	0.001	0.001	0.001	0.001
	(0.742)	(0.371)	(0.728)	(0.723)
INDEPENDENT	0.082*	0.077*	0.083*	0.080*
	(1.803)	(1.673)	(1.814)	(1.757)
BOARD	0.017	0.019	0.017	0.014
	(1.011)	(1.108)	(1.010)	(0.814)
_cons	0.973***	0.922***	0.966***	0.972***
	(7.849)	(7.572)	(7.771)	(7.872)
*N*	19,773	19,773	19,773	19,894
*R*^2^_a	0.019	0.056	0.019	0.020
Year/code	YES	YES	YES	YES

Values in parentheses are *t*-statistics; ***, **, * denote 1%, 5%, and 10% significance levels, respectively. All variables as previously defined.

### Further control of possible omitted variables

This study finds that the degree of strategic differentiation exacerbates firm leverage manipulation. Still, this finding may have endogeneity issues, specifically in this study, where risk-averse managers influence firm strategy on the one hand and choose to take risks in leverage manipulation on the other. At this point, the effect of strategic differentiation on leverage manipulation may be due to omitted variables. Although this is less likely because firm strategy is relatively stable and does not change as much as the individual manager would like, as Chandler suggests, “strategy, as a global and long-term plan, is the basis for a series of decisions affecting the firm, not a by-product of firm behavior.

However, to rule out the possibility of this omitted variable, this study, based on Li and Shi (2016), further controls for the effect of management’s risk appetite on leverage manipulation in the model. Specifically, the proportion of men among executives (DIRECTOR MANPRO), the proportion of men on the board of directors (EXECUTIVE MANPRO), and the average age of members of management governance (AVERAGEAGE) are added to the model, respectively. In addition, control variables for firm non-debt tax shield and ownership are added based on Zhai et al. (2021). The results, as shown in Table 12, show that the effect of strategic differentiation on leverage manipulation remains significantly positive at the 1% level after the inclusion of the above variables, indicating the robustness of this study’s findings.

**Table 12:** Control possible missing variables.

	(1)	(2)	(3)	(4)
	LEVM	ExpLEVM	ExpLEVMI	LEVM
SD	0.018***	0.020***	0.016***	
	(3.777)	(4.174)	(3.354)	
SD2				0.023***
				(5.223)
SIZE	−0.018***	−0.018***	−0.017***	−0.019***
	(−11.891)	(−11.782)	(−10.769)	(−12.069)
ROA	0.301***	0.300***	0.661***	0.305***
	(10.060)	(10.042)	(22.175)	(10.255)
CFO	−0.006	−0.008	−0.469***	−0.000
	(−0.223)	(−0.280)	(−17.084)	(−0.008)
GROWTH	0.002	0.003	−0.000	0.002
	(0.528)	(0.579)	(−0.076)	(0.536)
LEVB	0.142***	0.147***	0.139***	0.139***
	(12.565)	(12.939)	(12.648)	(12.379)
TOBIN	−0.003**	−0.003**	−0.004***	−0.004***
	(−2.518)	(−2.491)	(−3.043)	(−2.757)
INDEPENDENT	0.011	0.014	0.019	0.015
	(0.414)	(0.508)	(0.674)	(0.544)
BOARD	0.005	0.006	0.010	0.006
	(0.591)	(0.702)	(1.114)	(0.722)
AVERAGEAGE	−0.000	−0.001	−0.001	−0.000
	(−1.015)	(−1.082)	(−1.377)	(−1.008)
DIRECTOR MANPRO	−0.001	−0.000	−0.001	−0.001
	(−0.052)	(−0.019)	(−0.074)	(−0.059)
EXECUTIVE MANPRO	0.010	0.010	0.008	0.009
	(1.174)	(1.174)	(0.924)	(1.092)
NTDS	0.807***	0.783***	1.077***	0.793***
	(6.808)	(6.590)	(9.122)	(6.718)
SOE	−0.008**	−0.008**	−0.008**	−0.008**
	(−2.383)	(−2.324)	(−2.516)	(−2.437)
_cons	0.417***	0.410***	0.385***	0.417***
	(8.972)	(8.795)	(8.218)	(9.022)
*N*	19,256	19,256	19,256	19,373
*R*^2^_a	0.071	0.072	0.098	0.072
Industry	YES	YES	YES	YES
Year	YES	YES	YES	YES

Values in parentheses are *t*-statistics; ***, **, * denote 1%, 5%, and 10% significance levels, respectively. All variables as previously defined.

### Heckman two-stage model

To address the possible endogeneity problem, this study, based on Wang et al. (2019), uses the Heckman two-stage method to exclude sample selection bias. In the first stage, a Probit regression was conducted with the annual median of the strategic differentiation industry, defining the strategic differentiation dummy variable as the dependent variable. The model uses the mean value of strategic differentiation in the same industry year as an exclusion constraint variable for the strategic differentiation dummy variable and controls for firm size (SIZE), profitability (ROA), firm cash flow (CFO), firm growth (GROWTH), gearing (LEVB), market-to-book ratio (TOBIN), independent director proportion (INDEPENDENT), and board size (BOARD).

In the second stage, the above regression is re-run by adding the inverse Mills ratio (IMR) to the model (2). The results are shown in Table 13. After controlling for sample selection bias, the regression coefficient for the degree of strategic differentiation is still significantly positive at the 1% level, indicating the robustness of this study’s findings.

**Table 13:** Heckman two-step test.

	(1)	(2)	(3)	(4)
	LEVM	ExpLEVM	ExpLEVMI	LEVM
SD	0.015***	0.017***	0.013***	
	(3.333)	(3.750)	(2.830)	
SD2				0.021***
				(4.844)
SIZE	−0.004*	−0.004*	−0.006**	−0.005*
	(−1.800)	(−1.800)	(−2.324)	(−1.936)
ROA	−1.106***	−1.094***	−0.463**	−1.093***
	(−6.688)	(−6.587)	(−2.192)	(−6.603)
CFO	−0.224***	−0.225***	−0.618***	−0.217***
	(−5.786)	(−5.780)	(−13.402)	(−5.616)
GROWTH	0.026***	0.026***	0.018***	0.026***
	(5.094)	(5.083)	(3.285)	(5.084)
LEVB	0.499***	0.500***	0.418***	0.493***
	(11.625)	(11.600)	(7.203)	(11.505)
TOBIN	0.067***	0.067***	0.052***	0.067***
	(7.876)	(7.772)	(4.647)	(7.786)
INDEPENDENT	0.224***	0.225***	0.185***	0.226***
	(6.271)	(6.270)	(4.539)	(6.320)
BOARD	−0.120***	−0.118***	−0.089***	−0.118***
	(−6.996)	(−6.843)	(−4.116)	(−6.895)
IMR	1.293***	1.281***	1.018***	1.284***
	(8.745)	(8.631)	(5.234)	(8.690)
_cons	−1.219***	−1.212***	−0.893***	−1.210***
	(−6.239)	(−6.181)	(−3.559)	(−6.196)
*N*	19,773	19,773	19,773	19,892
*R*^2^_a	0.076	0.076	0.098	0.077
Industry	YES	YES	YES	YES
Year	YES	YES	YES	YES

Values in parentheses are *t*-statistics; ***, **, * denote 1%, 5%, and 10% significance levels, respectively. All variables as previously defined.

### PSM test

Based on Wang and Li (2020), this study utilizes the propensity score matching method to mitigate the possible omitted variables and the problem of sample self-selection. First, the strategic differentiation is divided into two groups according to whether it is greater than the industry’s annual median. Then, the high-degree group is assigned a value of 1, and the low-degree group is assigned a value of 0. Second, a logit regression model calculates the propensity score for firms to choose a high degree of strategic differentiation. The model includes all the control variables in (2). Again, one-to-one no-replay matching is used to find a corresponding matched sample for each firm with high strategic differentiation. Finally, regressions are performed using the paired completed samples, and the regression results are shown in Table 14.

**Table 14:** Propensity score-matched post-sample test.

	(1)	(2)	(3)	(4)
	LEVM	ExpLEVM	ExpLEVMI	LEVM
SD	0.012*	0.014**	0.010	
	(1.861)	(2.188)	(1.556)	
SD2				0.019***
				(3.298)
SIZE	−0.024***	−0.024***	−0.023***	−0.024***
	(−12.016)	(−11.947)	(−11.429)	(−12.175)
ROA	0.389***	0.388***	0.741***	0.392***
	(10.626)	(10.592)	(19.608)	(10.714)
CFO	−0.000	−0.003	−0.438***	0.004
	(−0.009)	(−0.090)	(−12.195)	(0.101)
GROWTH	−0.004	−0.004	−0.007	−0.004
	(−0.816)	(−0.765)	(−1.402)	(−0.847)
LEVB	0.157***	0.162***	0.158***	0.155***
	(10.912)	(11.215)	(10.938)	(10.844)
TOBIN	−0.005***	−0.005***	−0.006***	−0.005***
	(−3.168)	(−3.126)	(−3.596)	(−3.217)
INDEPENDENT	0.059	0.062*	0.062*	0.059
	(1.602)	(1.672)	(1.645)	(1.585)
BOARD	0.026**	0.027**	0.028**	0.025**
	(2.200)	(2.287)	(2.385)	(2.114)
_cons	0.490***	0.482***	0.469***	0.490***
	(8.146)	(7.991)	(7.748)	(8.215)
*N*	10,532	10,532	10,532	10,597
*R*^2^_a	0.072	0.072	0.098	0.072
Industry	YES	YES	YES	YES
Year	YES	YES	YES	YES

Values in parentheses are *t*-statistics; ***, **, * denote 1%, 5%, and 10% significance levels, respectively. All variables as previously defined.

Columns (1) and (2) show that the association between the degree of strategic differentiation and leverage manipulation (the basic leverage manipulation measure as well as the extended leverage manipulation measure under the direct method) are significantly positive at the 10% and 5% levels, respectively. On the other hand, column (3) shows that the coefficient on the association between the degree of strategic differentiation and leverage manipulation (leverage manipulation measure under the extended indirect method) is positive but not significant. Finally, column (4) shows that the coefficient on the association between the degree of strategic differentiation and leverage manipulation under the SD2 measure is significantly positive at the 1% level. Overall, each column’s results show that this study’s findings are robust.

### Other robustness tests

Finally, in this study, the results of the four ways of Tobit model estimation, replacing the dependent variable measure, replacing the independent variable measure, and changing the sample size, are placed in Table 15.

**Table 15:** Results of other robustness tests.

	(1)	(2)	(3)	(4)	(5)
	ExpLEVM	ExpLEVMI	LEVM	LEVM	LEVM
SD	0.018***	0.013***		0.007**	0.012**
SD2	(3.819)	(2.889)	0.022***	(2.121)	(2.454)
			(5.123)		
SIZE	−0.020***	−0.019***	−0.020***	−0.016***	−0.024***
	(−13.330)	(−12.448)	(−13.653)	(−14.222)	(−14.501)
ROA	0.265***	0.616***	0.270***	0.220***	0.317***
	(9.387)	(22.065)	(9.610)	(9.848)	(12.843)
CFO	0.032	−0.414***	0.040	0.124***	0.090***
	(1.260)	(−15.937)	(1.584)	(6.275)	(3.536)
GROWTH	0.003	−0.000	0.002	−0.003	0.001
	(0.595)	(−0.091)	(0.539)	(−1.233)	(0.342)
LEVB	0.143***	0.136***	0.135***	0.074***	0.173***
	(12.953)	(12.573)	(12.357)	(9.749)	(17.275)
TOBIN	−0.004***	−0.005***	−0.004***	−0.003***	−0.004***
	(−3.052)	(−3.687)	(−3.345)	(−3.213)	(−3.043)
INDEPENDENT	0.011	0.015	0.011	0.036*	0.013
	(0.404)	(0.543)	(0.423)	(1.716)	(0.409)
BOARD	0.006	0.009	0.006	0.007	0.009
	(0.633)	(1.065)	(0.651)	(1.099)	(0.963)
_cons	0.441***	0.421***	0.448***	0.335***	0.491***
	(10.374)	(9.913)	(10.616)	(11.365)	(10.318)
*N*	19,773	19,773	19,894	19,364	19,773
*R*^2^_a	0.068	0.093	0.068	0.081	/
Industry	YES	YES	YES	YES	YES
Year	YES	YES	YES	YES	YES

Values in parentheses are *t*-statistics; ***, **, * denote 1%, 5%, and 10% significance levels, respectively. All variables as previously defined.

Based on the extended XLT-LEVM method proposed by Xu et al. (2020) to measure firm leverage manipulation, the direct and indirect methods are used to calculate the estimated degree of leverage manipulation to mitigate the possible measurement error problem, and the regression results are shown in columns (1) and (2) of Table 15. Furthermore, the strategic differentiation (SD) is positively related to the leverage manipulation calculated using both the direct method (ExpLEVM) and the indirect method (ExpLEVMI), and both are significant at the 1% level, indicating that the results of this study are robust.

Based on the methodologies of previous studies, the measure of strategic differentiation adopted in the previous study uses selling expenses and intangible assets as approximate substitutes for advertising expenses and R&D expenses, which may lead to measurement errors in the variables and affect the reliability of the results. For this reason, this study excludes these two sub-indicators and uses the remaining four sub-indicators as a weighted average to calculate the degree of firm strategic differentiation (e.g., Yeh et al., 2015; Dong et al., 2021). The regression results are shown in column (3) of Table 15, and the coefficient of strategic differentiation (SD2) is positive and significant at the 1% level, indicating that the results of this study are robust.

Based on the method of Xu et al. (2021), samples with LEVM >1 are excluded to avoid the effect of extreme values when estimated using the XLT-LEVM model method, and the sample in 2007 is excluded to eliminate the effect of the implementation of the new accounting standards in 2007. The regression results are shown in column (4) of Table 15, where the association between strategic differentiation and leverage manipulation is significantly positive at the 5% level, which is consistent with the previous findings, indicating that the results of this study are robust.

Since the leverage manipulation is for downward leverage manipulation only, for this reason, the measured leverage manipulation variables take non-negative values and are truncated dependent variables. Considering this, this study employs Tobit truncation model regression for robustness testing (Zhai et al., 2021). The results are shown in column (5) of Table 15, in which the relationship between strategic differentiation and leverage manipulation remains significantly positive at the 5% level, indicating that the results of this study are robust.

## Research findings and policy recommendations

Strategic differentiation is an important part of strategic decisions. For example, firms with greater strategic differentiation may go for deleveraging in the form of leverage manipulation to meet their higher financing needs, reduce financing constraints, and cater to the call of policies. However, since the literature focuses more on the impact of strategic differentiation on the income statement (e.g., Liu and Lee, 2019), this study empirically examines the association between strategic differentiation and leverage manipulation based on the balance sheet of non-financial firms listed in Shanghai and Shenzhen A-shares from 2007 to 2020.

This study finds that the firm strategic differentiation is positively related to leverage manipulation, i.e., the greater strategic differentiation, the higher the likelihood of leverage manipulation by firms. Furthermore, the positive relationship between strategic differentiation and leverage manipulation is more significant in the group with higher short-term debt repayment pressure and in the group where the auditor does not have industry expertise. The mechanism analysis and empirical test results reveal that financing constraints are an important path through which strategic differentiation affects firm leverage manipulation.

The findings of this study suggest that the strategic differentiation of firms is an important internal trigger of their leverage manipulation, which is relevant for targeting the prevention of false deleveraging behavior of firms.

Specifically, the policy recommendations in this study have the following four aspects: government, regulatory, firm, and investment.

In terms of the government aspect, on the one hand, the government should provide targeted strategic management training and strategic management publicity and education services according to the development characteristics of local firms and guide them to choose a strategic model suitable for their development. On the other hand, the deleveraging policy focuses on the “real” rather than the “fast.” Deleveraging is necessary to put pressure on firms while encouraging them to improve their profitability and actively play the fundamental role of the market in resource allocation. Therefore, the government needs to focus on whether the deleveraging of firms is real and effective, further strengthen the review of large firm financing, release limited credit resources from firms with serious “leverage manipulation,” and allocate them to efficient and promising firms, and improve the efficiency of credit resource allocation in China.

Regarding the regulatory aspect, first, the relevant regulatory authorities should be precise in their policies to improve the effectiveness of regulatory policy implementation. The greater strategic differentiation, the higher the likelihood of firm leverage manipulation. Therefore, the SEC and other authorities must fully consider the impact of strategic differentiation on leverage manipulation and strengthen the monitoring of firms with a greater strategic positioning deviating from the conventional model. Second, the government should further regulate and guide firms to disclose this important non-financial information on strategy. The government should also encourage listed firms to disclose strategic information in more detail, raise the cost of violations arising from deviations in listed firms’ strategies, and reduce the behavior of firms that damage the value of the firm and the interests of investors or even disrupt the order of the capital market, due to excessive strategic differentiation. Third, the government should focus on balance sheet information manipulation, such as leverage manipulation, to prevent the flow of limited social resources to firms that conceal their high risk. Fourth, the Ministry of Finance and the CICPA, and other audit regulators should formulate strong policies to guide local firms to train and develop industry audit expertise and, on this basis, guide firms to hire auditors with industry expertise to improve the quality of firm information disclosure and protect investors’ interests through audit monitor.

Concerning the firm aspect, on the one hand, to achieve “real” deleveraging, firms must face up to the leverage problem, ease the pressure of deleveraging by improving their profitability, reduce the operational risks caused by the lack of firm coping capacity under the fierce market competition, and choose more ways to increase capital and retain profits to leverage prudently. On the other hand, to get long-term growth, firms need to focus on improving the quality of corporate governance. Using external auditors with specialized industry expertise to monitor firms can mitigate information asymmetry and facilitate access to lower-cost financing in the credit market.

In terms of the investment aspect, on the one hand, external investors should focus on non-financial information such as firm strategy to improve investment efficiency and reduce investment risks. On the other hand, strategic differentiation affects firm leverage manipulation. External investors can obtain a more reliable basis for investment decisions by assessing the value of a firm based on its strategic positioning by making forecasts. Since the greater strategic differentiation, the higher the possibility of firm leverage manipulation, external investors should pay attention to the potential risks that may arise from the strategic differentiation, maintain a cautious investment attitude toward firms that adopt industry differentiation strategies, interpret and analyze the information disclosed by firms comprehensively, and reduce the risk of information use. On the other hand, external investors should be rational in their investment choices. The positive association between strategic differentiation and firm leverage manipulation is more pronounced in firms with higher short-term debt repayment pressure and auditors without industry expertise. Therefore, investors need to dialectically understand the relationship between the firm strategic differentiation and leverage manipulation to identify risks accurately.

## Data availability statement

The original contributions presented in the study are included in the article/supplementary material, further inquiries can be directed to the corresponding author.

## Author contributions

FN, JW, and WS contributed to the conception and design of the study. FN organized the database. JW performed the statistical analysis and wrote the first draft of the manuscript. FN, JW, and WS wrote sections of the manuscript. All authors contributed to the manuscript revision, read, and approved the submitted version.

## Funding

This research was funded by Zhejiang Philosophy and Social Science Planning Project (grant number 23NDJC154YB).

## Conflict of interest

The authors declare that the research was conducted in the absence of any commercial or financial relationships that could be construed as a potential conflict of interest.

## Publisher’s note

All claims expressed in this article are solely those of the authors and do not necessarily represent those of their affiliated organizations, or those of the publisher, the editors and the reviewers. Any product that may be evaluated in this article, or claim that may be made by its manufacturer, is not guaranteed or endorsed by the publisher.

## Footnotes

^1^The data comes from the official website of the bank for International Settlements (IBS) at https://www.bis.org..

^2^The data is from the center of national balance sheet of the Chinese Academy of Social Sciences at http://www.nifd.cn/Activity/Future/14.
